# Protective role of *N*-acetyl-l-tryptophan against hepatic ischemia-reperfusion injury via the RIP2/caspase-1/IL-1β signaling pathway

**DOI:** 10.1080/13880209.2019.1617750

**Published:** 2019-06-11

**Authors:** Jianxin Wang, Shuna Yu, Jianguo Li, Huiting Li, Hongxin Jiang, Peilun Xiao, Yitong Pan, Jie Zheng, Li Yu, Jiying Jiang

**Affiliations:** a Department of Anatomy, Weifang Medical University, Weifang, China;; b Departments of Histology and Embryology, Weifang Medical University, Weifang, China;; c Morphology Laboratory of Weifang Medical University, Weifang, China;; d Department of Pathology, Weifang Medical University, Weifang, China

**Keywords:** Hepatic ischemia-reperfusion injury, N-acetyl-l-tryptophan, oxidative damaged

## Abstract

**Context:** Hepatic ischemia-reperfusion injury (HIRI) is a complex process observed during liver resection and transplantation. *N*-acetyl-l-tryptophan (l-NAT), an antagonist of neurokinin 1 receptor, has been used for the treatment of nausea and neurodegenerative diseases.

**Objective:** This study investigates the protective effect of l-NAT against HIRI and explores the potential underlying mechanisms.

**Materials and methods:** Adult male Sprague-Dawley (SD) rats were randomly divided into three groups: sham, I/R and I/R + l-NAT. HIRI model was generated by clamping the hepatic artery, portal vein and common bile duct with a microvascular bulldog clamp for 45 min, and then removing the clamp and allowing reperfusion for 6 h. BRL cells were exposed to 200 µM H_2_O_2_ with or without 10 µM l-NAT for 6 h.

**Results:** After l-NAT intervention, the structure of hepatic lobules was intact, and no swelling was noted in the cells. Furthermore, cell viability was found to be significantly enhanced when compared with the controls (*p* < 0.05). The mRNA and protein expression levels of serine-threonine kinase 2 (RIP2) and interleukin-1β (IL-1β) were significantly increased in the I/R and H_2_O_2_ groups when compared with the controls; however, these levels were significantly decreased after l-NAT intervention. Similarly, IL-1β activity and caspase-1 activity were significantly decreased in the H_2_O_2_ group when compared with the controls, after l-NAT intervention.

**Conclusions:** Our findings indicated that l-NAT may exert a hepatoprotective role in HIRI through inhibiting RIP2/caspase-1/IL-1β signaling pathway, which can provide evidence for l-NAT to be a potential effective drug against HIRI during clinical practice.

## Introduction

Hepatic ischemia-reperfusion injury (HIRI) is a complex process that occurs during partial hepatectomy, liver transplants, and other medical conditions (Golen et al. 2013; Woolbright and Jaeschke 2015; Kalogeris et al. 2016). It is currently believed that the essence of this process involves a neurogenic inflammatory response caused by the redelivery of blood circulation after organ ischemia. Thus, it is important to understand the mechanism by which these inflammatory reactions can be controlled in order to treat HIRI. Recent studies have demonstrated that Substance P (SP), an endogenous inflammatory medium, plays a key role in the neurogenic inflammatory response of many tissues and organs by combining with its specific receptor neurokinin 1 receptor (NK-1R) (Pintér et al. 2014; Corrigan et al. 2016; Vink et al. 2017). A number of experimental studies have evaluated the role of NK-1R antagonists in neurogenic inflammatory response; however, its role in HIRI remains unclear. Increasing evidence has shown the involvement of serine-threonine kinase 2 (RIP2)/caspase-1/interleukin (IL)-1β signaling pathway in cell injury mediated by the neurogenic inflammatory reaction. Nonetheless, NK-1R antagonist has become the focus in recent studies on neurogenic inflammation. Knockout of the NK-1R gene or NK-1R antagonist can relieve the neurogenic inflammatory response induced by hypoxic-ischemic brain damage (HIBD) (Jiang et al. 2011; Yu 2013), colitis (Reis et al. 2011), arthritis (Lam and Ng 2010; Makino et al. 2012), pancreatitis (Sakamoto et al. 2012) and respiratory inflammation (Yang et al. 2013). Hence, based on the findings of these studies, we speculated that the NK-1R antagonist might inhibit neurogenic inflammatory reaction after HIRI by inhibiting the RIP2/caspase-1/IL-1β signaling pathway. Thus, we investigated the expression of SP and NK-1R during HIRI and the effects of N-acetyl-l-tryptophan (l-NAT) on RIP2/caspase-1/IL-1β during the oxidative damage of BRL cells induced by H_2_O_2_. These results may not only yield novel targets for therapeutic intervention in HIRI but also provide a new direction to explore its mechanisms.

## Material and methods

### Animals and treatments

Male Sprague Dawley (SD) rats, weighing between 200 and 220 g, were purchased from Jinan Dion Shopping Jinan Pengyue Experimental Animal Center. Rats were randomly assigned to one of three groups: sham operation (sham) group, I/R group and I/R + l-NAT group (*n* = 6 per group). In the preliminary experiment, we found that 10 mg/kg l-NAT had less protective effect than 10 or 5 mg/kg l-NAT. Although the protective effects of 10 and 5 mg/kg were similar, we chose to use 5 mg/kg l-NAT in this experiment based on the principle of minimal use of clinical drugs. Sham group rats were subjected to the surgical procedures, but the clamp was not placed, and they were kept under anesthesia for an equivalent duration. l-NAT (5 mg/kg) or saline was administered i.p. 45 min before ischemia in the I/R + l-NAT and I/R groups. The HIRI model was induced according to previous literature (Wang et al. 2017). In preliminary experiments, we noted hepatic rope deformation, hepatocyte necrosis and massive neutrophil infiltration at 6 h post reperfusion, indicating that liver tissue damage was the most severe at that time, therefore rats were sacrificed 6 h after reperfusion. The Animal Ethics Committee of the University approved all working protocols.

### Cell culture and treatment

The rat hepatocyte BRL cell line was purchased from the Cell Bank of Type Culture Collection of the Chinese Academy of Sciences (Shanghai, China). The cells were divided into three groups: the control group, the H_2_O_2_ group, and the H_2_O_2_ +l-NAT group. The cell death model was established according to a previously described method (Huang et al. 2015). In brief, cells were challenged with 200 µM H_2_O_2_ with or without l-NAT for 18 h.

### Cell viability assay

Cell viability was determined using CCK-8 colorimetric assay (7sea-Biotech, Shanghai, China). BRL cells were seeded in a 96-well plate at a density of 2 × 10^8^/L. H_2_O_2_ (200 μM) was used to induce oxidative injury. In short, cells of H_2_O_2_ and H_2_O_2_+l-NAT groups were sowed in 96-well microplates overnight, then 100 μL CCK8/mL DMEM solution (5 mg/mL in PBS) (Logan, Utah, USA) was added. After 4 h of incubation in culture medium at 37 °C under 5% CO_2_ keep in a dark place. According to the product instructions, the OD (optical density) value at 450 nm was measured by a microplate reader. Three replicates were obtained from each group, and the mean was calculated. The cell viability was calculated using the following formula:

Cell viability (%)=(the OD of experimental group                    /the OD of control group) × 100%.

### Histopathological examination

Perfused livers were fixed with 4% paraformaldehyde for 24 h and then embedded in paraffin. For the detection of liver necrosis and apoptosis, formalin-fixed liver tissue sections were stained with hematoxylin and eosin (H&E) for the evaluation of necrosis.

### Immunohistochemistry

The cells were treated as previously described, fixed in 4% paraformaldehyde for 15 min, incubated in 0.3% Triton X-100-PBS for 10 min at room temperature and blocked with 5% goat serum for 30 min at 37 °C. The cells were subsequently incubated with an anti-RIP2 (1:200, Santa Cruz) at 4 °C overnight and incubated with FITC-conjugated secondary antibodies (1:150). These fluorescent slices were covered with mounting medium with DAPI and observed via confocal microscopy (Olympus FV500, Japan). The liver tissue of each group was fixed with 4% paraformaldehyde for 48 h, embedded in paraffin, and sliced (10 μm thick). Immunofluorescence staining of SP (1:200; Bioss, Beijing, bs-0065R) and NK1R (1:200; Bioss, Beijing, bs-0064R) was performed with routine methods. The reaction was followed by FITC-conjugated secondary antibodies (1:150) and covered with mounting medium with DAPI.

### Western blotting analysis

After l-NAT treatment, the total protein was extracted from the BRL cells and the rat liver tissue using protein extraction buffer (RIPA Beyotime Biotechnology, Shanghai, China). The cells were washed twice with ice-cold PBS (pH 7.4) and RIPA buffer (Solarbio, Beijing, China). Proteins were subjected to SDS-PAGE with a 10% running gel and then transferred to a PVDF membrane. The samples were incubated with the following antibodies: anti-NF-κB (1:500, Bioss, Beijing, bs-0465R); anti-TLR-4 (1:500, Bioss, Beijing, bs-1021R); anti-RIP2 (1:500, Santa Cruz); and anti-GAPDH (1:2000, Proteintech, 10494-1-AP), respectively. The details for the analysis have been described previously (Huang et al. 2015). Western blot images were analyzed using ImageJ software (National Institutes of Health, Bethesda, MD). GAPDH was used as the internal reference protein.

#### Quantitative real-time PCR (qRT-PCR)

Following the l-NAT treatment, TRIzol agentia (Life) was used to extract the total RNA of the BRL cells and the rat liver tissues according to the manufacturer’s protocol, and the total RNA was reverse transcribed into cDNA using the Prime Script RT Master Mix (Takara, Japan) as instructed. Real-time PCR was executed using 5 µM of 2× SYBR Green Master (Indianapolis, IN), 0.6 µL of each primer and 1 µL of cDNA sample and H_2_O to a final volume of 10 µL. PCR was performed as previously reported (Sakamoto et al. 2012). In brief, the cDNA was pre-denatured at 94 °C for 3 min, annealed at 60 °C for 45 s, and amplified for 39 cycles; the total mRNA for each gene was standardized by GAPDH, and the relative levels were computed using the 2^−ΔΔCT^ method as previously reported (Yu 2013). The primer (Sangon Biotech, Shanghai, China) sequences are shown in Table 1.

**Table 1.** Nucleotide sequences of primers used for qRT-PCR.

Gene	Sequence
Caspase-1	F:5′-TCGAGCCAGAATGAGGACTG-3′
	R:5′-GATGATGTTGGCAGCAATGG-3′
IL-1β	F:5′-CTGGTGCATTCTGACCTTGC-3′
	R:5′-GGTCCATCTCCTTGGTCTGC-3′
RIP2	F:5′-TCGTGCTCCTTGACTGTGAT-3′
	R:5′-CGGTCCTTGTAGGTTTGGTG-3′
GAPDH	F:5′-TGATTCTACCCACGGCAAGTT-3′
	R:5′-TGATGGGTTTCCCATTGATGA-3′

### ELISA analysis

The levels of IL-1β in the culture medium were measured using rat ELISA kits (NeoBioscience, Shanghai, China) according to the manufacturer’s instructions. The plates were read at 450 nm using a microplate reader (Thermo, Shanghai, China).

### Caspase-1 activity assay

Liver samples and BRL cells collected and rinsed with PBS, lysed in lysis buffer for 15 min on ice, then centrifuged at 20,000 *g* for 10 min at 4 °C. Caspase-1 activity in the supernatant was assayed with commercially available kits (Beyotime Biotechnology, Shanghai, China), according to the manufacturer’s instructions. All experiments were performed in triplicate.

### Statistical analysis

All *in vitro* experiments were repeated three times, and the *in vivo* experiments were repeated five times. All data are presented as the mean ± SD values. The statistical analyses of the data were performed using a one-way analysis of variance (ANOVA) followed by the least significant difference (LSD) test with Statistical Package for the Social Sciences (SPSS, version 17.0) software. Furthermore, *p* < 0.05 were considered significant.

## Results

### l-NAT significantly alleviated H_2_O_2_-induced damage in BRL cells

**Figure 1.**
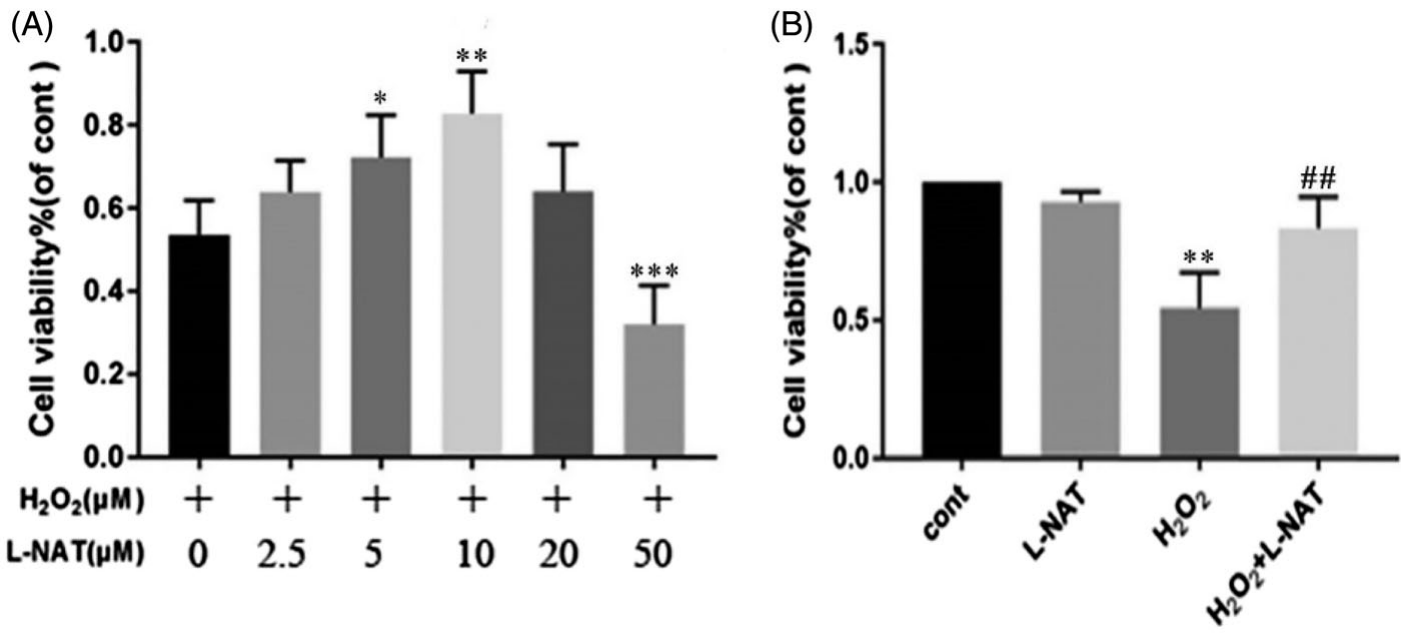
Cell viability of BRL cells measured by CCK-8 assay. The results are presented as a percentage of the control. Cell viability of BRL cells following different concentrations of l-NAT exposure was measured by CCK-8 assay. BRL cells were treated with 200 μM H_2_O_2_ with or without a series of concentrations of l-NAT (0, 2.5, 5, 10, 20 or 50 μM) for 18 hours. *p < 0.05, **p < 0.01, ***p < 0.001 versus untreated group and ^##^*p* < 0.01 compared with related H_2_O_2_ group or I/R group.

The protective effect of l-NAT on H_2_O_2_-induced oxidative damage in BRL cells was investigated by CCK-8 staining. Cell viability was found enhanced with the increase in l-NAT concentration, reaching a peak of 10 μM and beginning its descent at 20 μM. The cell activity was lower than that in the normal group at 50 μM (0.69 ± 0.334 versus 0.401 ± 0.136, *p* < 0.05) (Figure 1).

### l-NAT protects the liver against HIRI *in vivo* in the rat model

**Figure 2.**
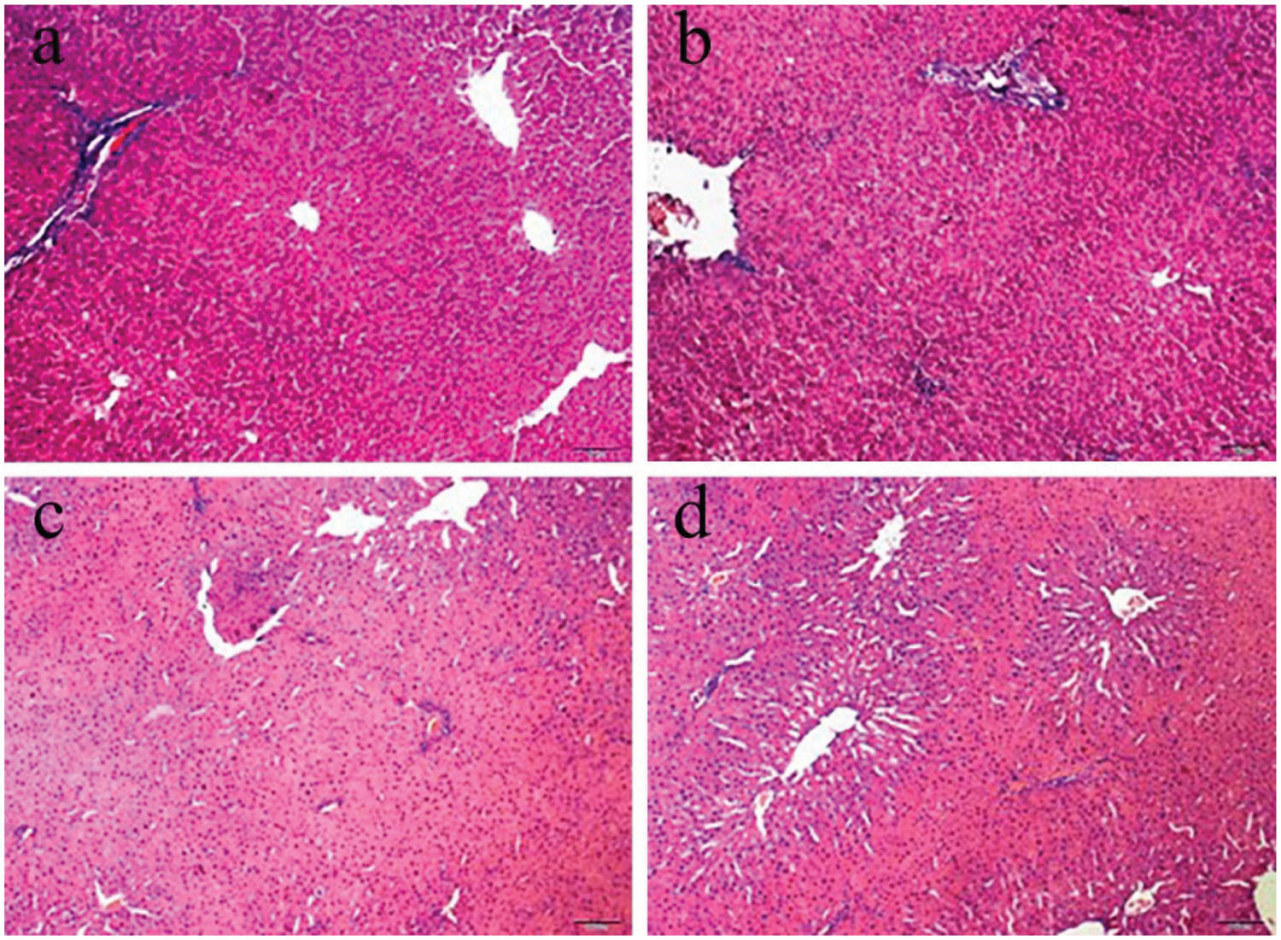
Liver damage was analyzed by liver histology (representative H&E staining; original magnifications, 100× magnification). (a) Sham, (b) l-NAT, (c) I/R, (d) I/R + l-NAT.

To investigate the effects of l-NAT on HIRI, l-NAT was injected via intraperitoneal injection at 10 mg/kg, 0.5 h before ischemia. The hepatocytes in the sham group were regularly arranged and presented with a normal structure with no damage or loss, whereas in the I/R group, the cells were disorderly arranged, swollen, and ruptured. l-NAT significantly attenuated the changes in the hepatocytes (Figure 2).

### Effects of l-NAT on RIP2 expression *in vitro* and *in vivo*

Research has shown that RIP2, a stress-induced upstream modulator of procaspase-1 apoptotic activation, may cause oligomeric activation of procaspase-1 (Manon et al. 2007). Therefore, to study the underlying mechanism by which l-NAT exerts its hepatoprotective effect, we used both the H_2_O_2_-triggered BRL cell injury model and the rat HIRI model to detect the expression of RIP2.

**Figure 3.**
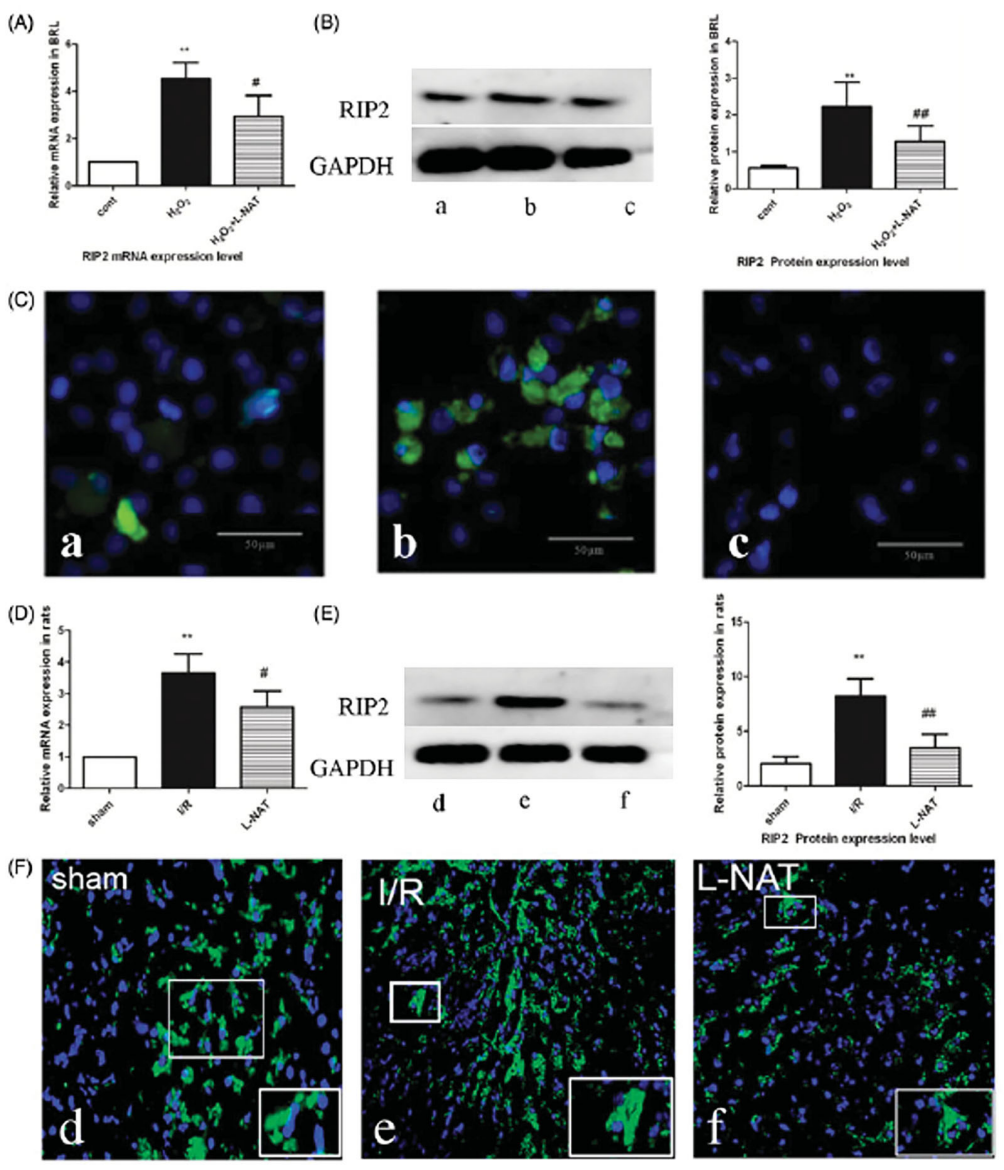
Changes in the expression of RIP2 after HIRI. (A) Relative mRNA levels of RIP2 in BRL cells. Detected by quantitative qRT-PCR (*n* = 3). (B) Western blot analysis of RIP2 protein expression in BRL cells. (C) Immunofluorescence staining (200X) in BRL cells. (a) Cont, (b) H_2_O_2_, (c) H_2_O_2+_l-NAT. (D) Relative mRNA levels of RIP2 in rat liver tissues detected using quantitative qRT-PCR (*n* = 3). (E) Western blot analysis of RIIP2 protein expression in rat liver tissues. (F) Immunofluorescence staining (200×) in rat liver tissues (d) sham, (e) I/R, (f) l-NAT. Data are mean ± SD; **p < 0.01, *p < 0.05 compared with the related Cont group or sham group, ^##^*p* < 0.01, ^#^*p* < 0.05 compared with related H_2_O_2_ group or I/R group.

*In vitro*, the immunofluorescent expression levels of RIP2, caspase-1 and IL-1β were significantly increased in the H_2_O_2_ group when compared with the control group (*p* < 0.05; Figure 3(C)a–c). However, the expression levels of RIP2, caspase-1 and IL-1β were significantly reduced following l-NAT administration when compared with the H_2_O_2_ group (*p* < 0.05; Figure 3(D–F)). qRT-PCR results revealed a dramatic increase in the mRNA expression levels of RIP2 in the H_2_O_2_ group when compared with the control group (*p* < 0.05; Figure 3(A)). However, treatment with l-NAT (10 μM) significantly reduced RIP2 expression levels when compared to those in the H_2_O_2_ group. Similarly, western blot analysis also showed a significant increase in the protein expression levels of RIP2 in the H_2_O_2_ group (*p* < 0.05; Figure 3(B,C)) relative to the controls. In contrast, treatment with l-NAT (10 μM) significantly reduced RIP2 levels when compared with the H_2_O_2_ group (*p* < 0.05; Figure 3(B)). Thus, the above results indicate that l-NAT protected H_2_O_2_-induced injury by inhibiting the relative expression levels of RIP2.

*In vivo*, after l-NAT (10 mg/kg) was used, RIP2, caspase-1, and IL-1β levels in the liver tissues of rats were detected via immunofluorescence staining. The results showed that the expression levels of RIP2, caspase-1 and IL-1β were higher in the I/R group when compared with the sham group (Figure 3(F)). Both qRT-PCR and western blot analyses showed significant increases in RIP2 expression levels at the mRNA and protein levels, respectively, in the I/R group (Figure 3(D,E)) when compared with the sham group. However, treatment with l-NAT (100 mg/kg body weight) reduced the expression levels (Figure 3(D–F)) suggesting that RIP2 may be involved in the hepatoprotective effect of l-NAT after HIRI.

### Effect of l-NAT on caspase-1 expression *in vitro* and *in vivo*

**Figure 4.**
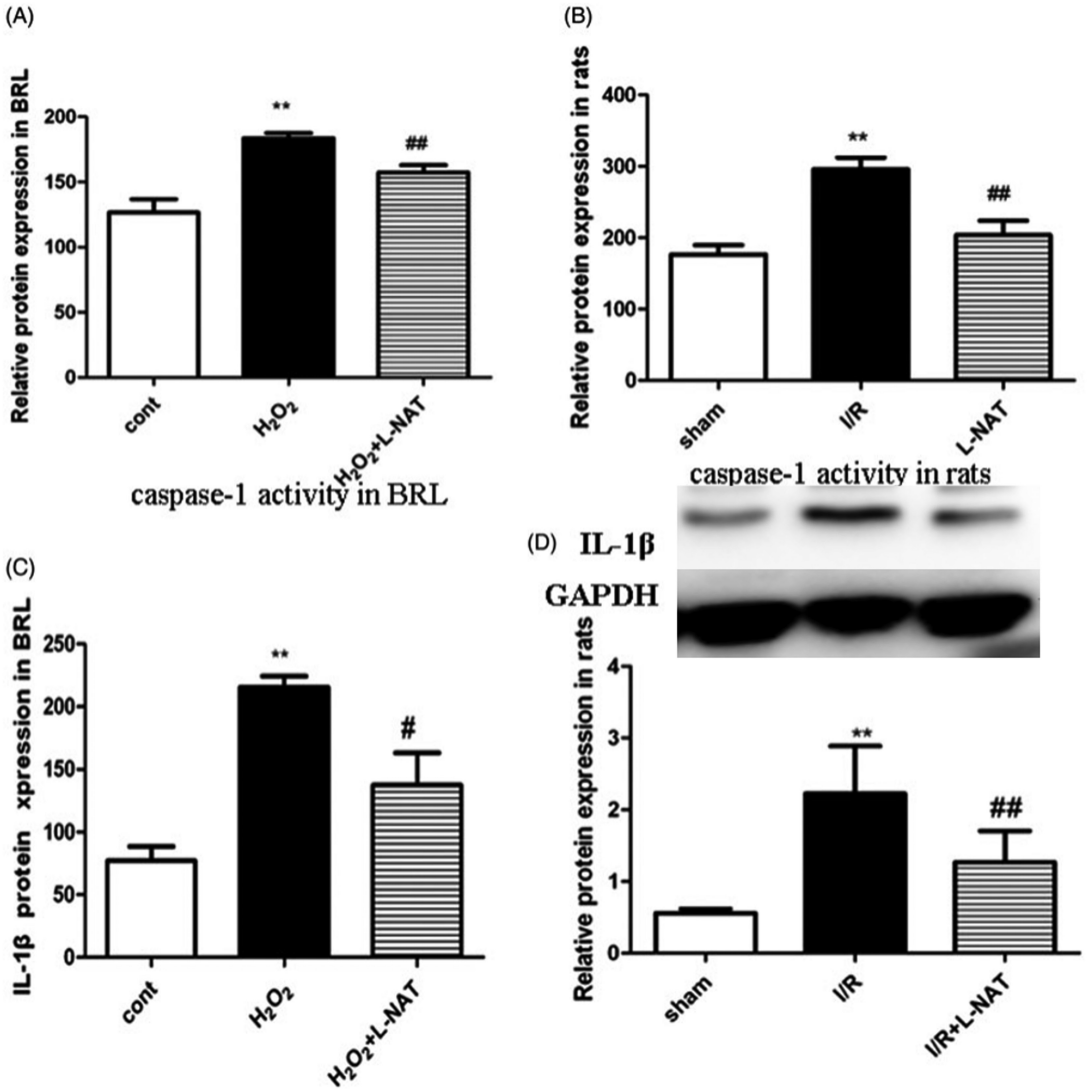
Changes of Caspase-1 viability and IL-1β expression *in vitro* and *in vivo*. (A) Relative Caspase-1 viability in BRL cells. (*n* = 3). (B) Relative Caspase-1 viability in rat liver tissues. (*n* = 3). Data are mean ± SD; **p < 0.01, *p < 0.05 compared with the related CON group or sham group, ^##^*p* < 0.01, ^#^*p* < 0.05 compared with related H_2_O_2_ group or I/R group. (C) Relative protein levels of IL-1β in BRL cells detected using ELISA (*n* = 3). (D) Western blot analysis of IL-1β protein expression in rat liver tissues (*n* = 3).

To evaluate further the protective role of l-NAT, caspase-1 activity was assessed using the enzyme cleavage assay *in vitro* and *in vivo*. Caspase-1 activity in the BRL cells was higher in the H_2_O_2_ group when compared with the control group (*p* < 0.05; Figure 4(A)). Furthermore, a significant reduction in caspase-1 activity was noted following treatment with l-NAT when compared to that in the H_2_O_2_ group (*p* < 0.05; Figure 4(A)). A similar trend was observed *in vivo,* caspase-1 activity in the liver tissues was significantly increased in the I/R group when compared with the sham group (*p* < 0.05; Figure 4(B)). However, treatment with l-NAT (10 mg/kg) resulted in a significant decrease in caspase-1 activity (*p* < 0.05; Figure 4(B)), indicating that l-NAT could reduce caspase-1 activity after HIRI.

### Effects of l-NAT on IL-1β expression *in vitro* and *in vivo*

ELISA was used to investigate the concentration of IL-1β in the culture medium. As shown in Figure 4(C), the BRL cells subjected to H_2_O_2_ (200 μM) showed a dramatic increase in IL-1β levels when compared with the control group (*p* < 0.05). l-NAT (10 μM) treatment resulted in a significant decrease in IL-1β expression levels when compared with the H_2_O_2_ group (*p* < 0.05). Likewise, IL-1β expression levels were significantly increased in the I/R group *in vivo* when compared with the controls, and decreased in the l-NAT group relative to the H_2_O_2_ group (*p* < 0.05; Figure 4(D)). To sum up, this study demonstrates that l-NAT could inhibit the expression and secretion of IL-1β induced by HIRI.

## Discussion

In this study, we used both *in vivo* (rat HIRI) and *in vitro* (H_2_O_2_-induced BRL cells and oxidatively damaged cells) models to study the effect of l-NAT on HIRI. l-NAT administration was proved to be a simple and effective means to minimize HIRI in the rats. More importantly, the data showed that l-NAT alleviates HIRI by affecting the activity of the RIP2/caspase-1/IL-1β signaling pathway. HIRI, a condition that seriously affects postoperative recovery, has become an urgent clinical problem owing to the increase in the frequency of transplantations and resection surgical procedures in recent years. Direct injury induced by ischemia-hypoxia causes the damaged hepatocytes to release damage-associated molecular patterns (DAMPs), which become active in response to molecular stimuli. The DAMPs activate hepatocytes and pattern recognition receptors on nonparenchymal surfaces, initiating local or systemic immune responses and mediating further injury (Maharana et al. 2014). Thus, natural recognition receptors and their signaling pathways play a key role in HIRI. All toll-like receptors (TLRs) and NODs are pattern recognition receptors that recognize the extracellular risk signals outside the cell and extracellular risk signals inside the cell, respectively. Additional research has shown that the inflammatory response induced by both TLRs and NODs can accelerate the nuclear translocation of NF-κB through IKKγ. Taken together, the two signaling pathways directly join at IKKγ; RIP2, the upstream molecule of IKKγ, is a common downstream molecule of the TLRs and NODs signaling pathways (Manon et al. 2007; Kersse et al. 2011; Fridh and Rittinger 2012; Cai et al. 2013; Jeong et al. 2013; Heymann et al. 2014). The RIP2 protein can interact with CARD-CARD in the CARD region of the NOD protein. After binding to NOD, RIP2 interacts with IKKγ, the regulatory subunit of the inhibitory κB kinase, which activates the NF-κB pathway to promote the expression of a series of pro-inflammatory cytokines such as TNF-α, IL-1, IL-6 and ICAM-1. Furthermore, caspase-1 plays a key role in the inflammatory pathways by processing pro-IL-1β into the active cytokine, mature. In particular, the processing of procaspase-1 to IL-1β is accelerated by the association of the zymogen with RIP2. Based on these facts, we speculated that the RIP2/caspase-1/IL-1β signaling might play a role in the inflammatory response induced by HIRI.

Kersse et al. (2011) reported that the RIP2/caspase-1 axis plays an important role in cell death in lethally challenged cerebral ischemia-reperfusion injury, Huntington’s disease, cerebral cortical neurons, the striatum, NSC-34 cells, ST14A cells and HeLa cells induced by H_2_O_2_, OGD, TNF-α/CHX, and other noxious, which is aggravated by the overexpression and ameliorated by the down expression of RIP2 and caspase-1. However, the changes in the RIP2 signaling pathway in HIRI have not been reported so far. In the current study, the expression levels of pro-inflammatory factors such as RIP2 were dramatically decreased after the development of HIRI, suggesting that l-NAT protects the liver by inhibiting the expression of RIP2. Research has shown that RIP2 and procaspase-1 interact via their homologous caspase recruitment domains (also referred to as CARDIAK and RICK), which cause oligomeric activation of procaspase-1 to from mature caspase-1 (Fridh and Rittinger 2012; Szabo and Petrasek 2015). The latter could further cleave pro-IL-1β into mature IL-1β, which is an important cytokine that promotes inflammatory injury during hepatic I/R. The activation of the caspase-1/IL-1β pathway promoted high mobility group box-1 protein induction to facilitate a TLR4-dependent inflammatory phenotype leading to I/R hepatocellular damage. IL-1β has been shown to stimulate NF-κB activation, hepatic neutrophil accumulation, and the production of pro-inflammatory cytokines (Grady et al. 2000; Tsung et al. 2005). Consistent with the results of another study (Jiang et al. 2014), our data confirmed that the anti-inflammatory effects were partly due to the inhibition of the mRNA and protein expressions of the pro-inflammatory cytokine IL-1β.

The role of SP-mediated neurogenic inflammation in liver disease has also been reported. Bang et al. (2003, 2004) discovered through a series of findings that various types of liver cells can express NK-1R, and that exogenous SP can promote hepatocyte damage. Furthermore, NK1R knockout or NK1R antagonists could reduce acute hepatic injury induced by CD95 and TNF-α. l-NAT, as an antagonist of NK-1R, has been approved by the FDA for the treatment of nausea, vomiting, shock and neurodegenerative diseases. It has been reported that the levels of SP and NK-1R were upregulated in patients and animal models of pancreatitis, arthritis, bronchial asthma, rheumatism, colitis and other inflammatory lesions. However, the role of SP/NK-1R in HIRI, and whether NK1R antagonists could ameliorate the extent of hepatocyte injury have not been reported so far. Interestingly, treatment with l-NAT improved viability and survival of BRL cells in the current study and attenuated the inflammatory response by down-regulating RIP2/caspase-1/IL-1β signaling both *in vitro* and *in vivo*. Therefore, we believe that the protective effects of l-NAT on hepatocytes might be mediated, at least in part, by the RIP2/caspase-1/IL-1β signaling pathway.

The main limitation of this study is that we did not knockout or block the RIP2 gene in the BRL cells or rats. Therefore, we could provide more direct evidence that the protective effect of l-NAT against HIRI is RIP2-mediated. However, we intend to explore this in future experiments and investigate the detailed mechanism of l-NAT protection.

## Conclusions

In this study, alterations in the SP/NK-1R system were investigated in an *in vivo* HIRI model, while the influence of l-NAT on hepatic function, inflammation, and activation of the RIP2/caspase-1/IL-1β signaling pathway was investigated in an *in vitro* H_2_O_2_-induced hepatocyte injury model. l-NAT lowered the relative gene expression levels of RIP2, TLR4 and NF-κB, and decreased caspase-1 and IL-1β activation, thereby implying that l-NAT alleviates HIRI by affecting the RIP2/caspase-1/IL-1β signaling pathway. Our findings indicated that l-NAT may exert a hepatoprotective role in HIRI through inhibiting RIP2/caspase-1/IL-1β signaling pathway, which can be a potential effective drug against HIRI during clinical practice.

## References

[CIT0001] Bang R, Biburger M, Neuhuber WL, Tiegs G. 2003. Neurokinin-1 receptor antagonists protect mice from CD95- and tumor necrosis factor-alpha-mediated apoptotic liver damage. J Pharmacol Exper Ther. 308:1174–1180. 14617692 10.1124/jpet.103.059329

[CIT0002] Bang R, Sass G, Kiemer AK, Vollmar AM, Neuhuber WL, Tiegs G. 2003. Neurokinin-1 receptor antagonists CP-96,345 and L-733,060 protect mice from cytokine-mediated liver injury. J Pharmacol Exp Ther. 305:31–39. 12649350 10.1124/jpet.102.043539

[CIT0003] Cai X, Du J, Liu Y, Xia W, Liu J, Zou M, Wang Y, Wang M, Su H, Xu D. 2013. Identification and characterization of receptor-interacting protein 2 as a TNFR-associated factor 3 binding partner. Gene. 517:205–211. 23333941 10.1016/j.gene.2012.12.026

[CIT0004] Corrigan F, Vink R, Turner RJ. 2016. Inflammation in acute CNS injury: a focus on the role of substance P. Br J Pharmacol. 173:703–715. 25827155 10.1111/bph.13155 PMC4742293

[CIT0005] Fridh V, Rittinger K. 2012. The tandem CARDs of NOD2: intramolecular interactions and recognition of RIP2. PLoS One. 7:e34375. 22470564 10.1371/journal.pone.0034375 PMC3314614

[CIT0006] Golen RF, Reiniers MJ, Olthof PB, Gulik TM, Heger M. 2013. Sterile inflammation in hepatic ischemia/reperfusion injury: present concepts and potential therapeutics. J Gastroenterol Hepatol. 28:394–400. 23216461 10.1111/jgh.12072

[CIT0007] Grady EF, Yoshimi SK, Maa J, Valeroso D, Vartanian RK, Rahim S, Kim EH, Gerard C, Gerard N, Bunnett NW, et al. 2000. Substance P mediates inflammatory oedema in acute pancreatitis via activation of the neurokinin-1 receptor in rats and mice. Br J Pharmacol. 130:505–512. 10821777 10.1038/sj.bjp.0703343 PMC1572103

[CIT0008] Heymann MC, Winkler S, Luksch H, Flecks S, Franke M, Ruß S, Ozen S, Yilmaz E, Klein C, Kallinich T, et al. 2014. Human procaspase-1 variants with decreased enzymatic activity are associated with febrile episodes and may contribute to inflammation via RIP2 and NF-κB signaling. J Immunol. 192:4379–4385. 24706726 10.4049/jimmunol.1203524

[CIT0009] Huang H, Tohme S, Al-Khafaji AB, Tai S, Loughran P, Chen L, Wang S, Kim J, Billiar T, Wang Y, et al. 2015. Damage-associated molecular pattern-activated neutrophil extracellular trap exacerbates sterile inflammatory liver injury. Hepatology. 62:600–614. 25855125 10.1002/hep.27841 PMC4515210

[CIT0010] Jeong YJ, Kim CH, Kim JC, Oh SM, Lee KB, Park JH, Kim DJ. 2013. RIP2/RICK-dependent cytokine production upon *Yersinia enterocolitica* infection in macrophages with TLR4 deficiency. Scand J Immunol. 78:401–407. 23952047 10.1111/sji.12100

[CIT0011] Jiang J, Yu S, Jiang Z, Liang C, Yu W, Li J, Du X, Wang H, Gao X, Wang X. 2014. *N*-acetyl-serotonin protects HepG2 cells from oxidative stress injury induced by hydrogen peroxide. Oxid Med Cell Longev. 12:1–15. 10.1155/2014/310504 PMC4074966 25013541

[CIT0012] Jiang JY, Jiang GL, Li L, Zhao JL, Shi CX, Yu SN, Qi AD. 2011. Protective effect of *N*-acetyl-l-tryptophan in cerebral ischemia/hypoxia injury. Acta Anatomica Sinica. 42:451–455.

[CIT0013] Kalogeris T, Baines CP, Krenz M, Korthuis RJ. 2016. Ischemia/reperfusion. Compr Physiol. 7:113–170. 28135002 10.1002/cphy.c160006 PMC5648017

[CIT0014] Kersse K, Lamkanfi M, Bertrand MJ, Vanden Berghe T, Vandenabeele P. 2011. Interaction patches of procaspase-1 caspase recruitment domains (CARDs) are differently involved in procaspase-1 activation and receptor-interacting protein 2 (RIP2)-dependent nuclear factor κB signaling. J Biol Chem. 286:35874–35882. 21862576 10.1074/jbc.M111.242321 PMC3195576

[CIT0015] Lam FF, Ng ES. 2010. Substance P and glutamate receptor antagonists improve the anti-arthritic actions of dexamethasone in rats. Br J Pharmacol. 159:958–969. 20128799 10.1111/j.1476-5381.2009.00586.x PMC2829221

[CIT0016] Maharana J, Patra MC, De BC, Sahoo BR, Behera BK, De S, Pradhan SK. 2014. Structural insights into the MDP binding and CARD-CARD interaction in zebrafish (*Danio rerio*) NOD2: a molecular dynamics approach. J Mol Recogn. 27:260–275. 10.1002/jmr.2357 24700593

[CIT0017] Makino A, Sakai A, Ito H, Suzuki H. 2012. Involvement of tachykinins and NK1 receptor in the joint inflammation with collagen type II-specific monoclonal antibody-induced arthritis in mice. J Nippon Med School. 79:129–138. 10.1272/jnms.79.129 22687356

[CIT0018] Manon F, Favier A, Núñez G, Simorre J, Cusack S. 2007. Solution structure of NOD1 CARD and mutational analysis of its interaction with the CARD of downstream kinase RICK. J Mol Biol. 365:160–174. 17054981 10.1016/j.jmb.2006.09.067

[CIT0019] Pintér E, Pozsgai G, Hajna Z, Helyes Z, Szolcsányi J. 2014. Neuropeptide receptors as potential drug targets in the treatment of inflammatory conditions. Br J Clin Pharmacol. 77:5–20. 23432438 10.1111/bcp.12097 PMC3895342

[CIT0020] Reis RC, Brito HO, Fraga D, Cabrini DA, Zampronio AR. 2011. Central substance P NK₁ receptors are involved in fever induced by LPS but not by IL-1β and CCL3/MIP-1α in rats. Brain Res. 1384:161–169. 21303668 10.1016/j.brainres.2011.02.001

[CIT0021] Sakamoto T, Kamijima M, Miyake M. 2012. Neurogenic airway microvascular leakage induced by toluene inhalation in rats. Eur J Pharmacol. 685:180–185. 22554773 10.1016/j.ejphar.2012.04.035

[CIT0022] Szabo G, Petrasek J. 2015. Inflammasome activation and function in liver disease. Nat Rev Gastroenterol Hepatol. 12:387–400. 26055245 10.1038/nrgastro.2015.94

[CIT0023] Tsung A, Sahai R, Tanaka H, Nakao A, Fink MP, Lotze MT, Yang H, Li J, Tracey KJ, Geller DA, et al. 2005. The nuclear factor HMGB1 mediates hepatic injury after murine liver ischemia-reperfusion. J Exp Med. 201:1135–1143. 15795240 10.1084/jem.20042614 PMC2213120

[CIT0024] Vink R, Gabrielian L, Thornton E. 2017. The role of substance P in secondary pathophysiology after traumatic brain injury. Front Neurol. 8:304. 28701994 10.3389/fneur.2017.00304 PMC5487380

[CIT0025] Wang S, Jiang H, Li X, Hu M, Hai Y, Ming Z, Yu S, Wang J, Jiang J. 2017. Protective effect of N-acetyl-l-tryptophan on intestinal damage after rat hepatic ischemia reperfusion. Chongqing Med. 46:3748–3751.

[CIT0026] Woolbright BL, Jaeschke H. 2015. Sterile inflammation in acute liver injury: myth or mystery? Expert Rev Gastroenterol Hepatol. 9:1027–1029. 26186639 10.1586/17474124.2015.1060855 PMC4613762

[CIT0027] Yang Y, Yan M, Zhang H, Wang X. 2013. Substance P participates in immune-mediated hepatic injury induced by concanavalin A in mice and stimulates cytokine synthesis in Kupffer cells. Exp Therap Med. 6:459–464. 24137208 10.3892/etm.2013.1152 PMC3786810

[CIT0028] Yu S. 2013. *N*-acetyl-l-tryptophan alleviates the apoptosis induced by H_2_O_2_ of mice PHNs. Basic Clinl Med. 33:423–428.

