# Correction

**DOI:** 10.1080/13880209.2025.2444094

**Published:** 2024-12-22

**Article title**: Protective role of *N*-acetyl-l-tryptophan against hepatic ischemia-reperfusion injury via the RIP2/caspase-1/IL-1β ­signaling pathway

**Authors**: Jianxin Wang, Shuna Yu, Jianguo Li, Huiting Li, Hongxin Jiang, Peilun Xiao, Yitong Pan, Jie Zheng, Li Yu and Jiying Jiang

**Journal**: *Pharmaceutical Biology*

**Bibliometrics**: Volume 57, Number 1, pages 385–391

**DOI**: https://doi.org/10.1080/13880209.2019.1617750

This paper contains an error as the bands provided in the main text are blurry and cannot clearly indicate the upward trend of the IR group in the western blot bands in Figure 4D when the above-mentioned article was first published online.

The corrected Figure 4 has been displayed below and the article has been republished online.

**Figure 4.**
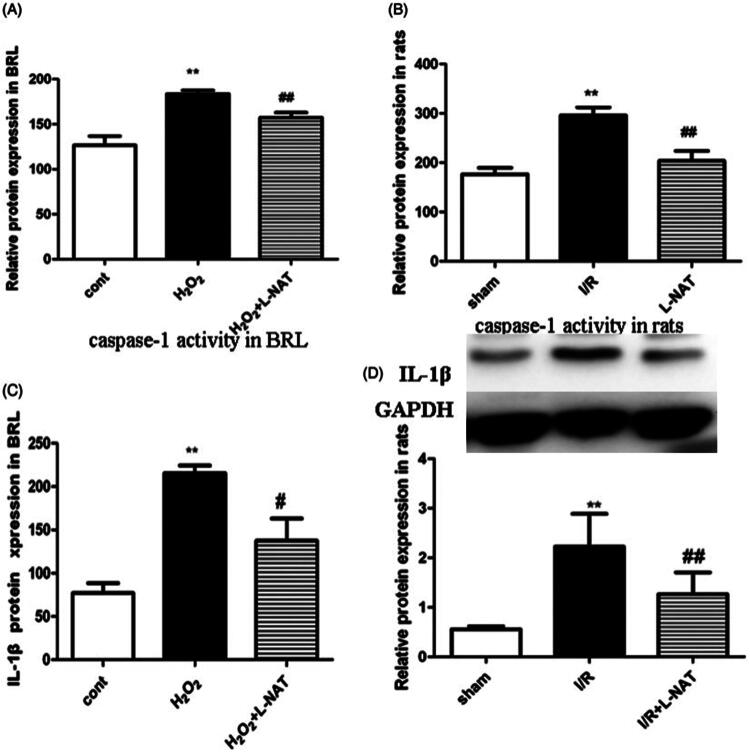
Changes of Caspase-1 viability and IL-1β expression *in vitro* and *in vivo*. (A) Relative Caspase-1 viability in BRL cells. (*n* = 3). (B) Relative Caspase-1 viability in rat liver tissues. (*n* = 3). Data are mean ± SD; **p < 0.01, *p < 0.05 compared with the related CON group or sham group, ^##^*p* < 0.01, ^#^*p* < 0.05 compared with related H_2_O_2_ group or I/R group. (C) Relative protein levels of IL-1β in BRL cells detected using ELISA (*n* = 3). (D) Western blot analysis of IL-1β protein expression in rat liver tissues (*n* = 3).

